# Targeted disruption of the *Kcnj5* gene in the female mouse lowers aldosterone levels

**DOI:** 10.1042/CS20171285

**Published:** 2018-01-16

**Authors:** Iris Hardege, Lu Long, Raya Al Maskari, Nicola Figg, Kevin M. O’Shaughnessy

**Affiliations:** 1 Department of Medicine, EMIT Division, Addenbrooke’s Hospital, Hills Road, Cambridge, U.K.; 2 Department of Medicine, CVD Division, Addenbrooke’s Hospital, Hills Road, Cambridge, U.K.

**Keywords:** aldosterone, GIRK4 K channel, KCNJ5 gene, knockout mice, RNAseq

## Abstract

Aldosterone is released from adrenal zona glomerulosa (ZG) cells and plays an important role in Na and K homoeostasis. Mutations in the human inwardly rectifying K channel CNJ type (*KCNJ*) *5* (*KCNJ5*) gene encoding the G-coupled inwardly rectifying K channel 4 (GIRK4) cause abnormal aldosterone secretion and hypertension. To better understand the role of wild-type (WT) GIRK4 in regulating aldosterone release, we have looked at aldosterone secretion in a *Kcnj5* knockout (KO) mouse. We found that female but not male KO mice have reduced aldosterone levels compared with WT female controls, but higher levels of aldosterone after angiotensin II (Ang-II) stimulation. These differences could not be explained by sex differences in aldosterone synthase (*Cyp11B2*) gene expression in the mouse adrenal. Using RNAseq analysis to compare WT and KO adrenals, we showed that females also have a much larger set of differentially expressed adrenal genes than males (395 compared with 7). Ingenuity Pathway Analysis (IPA) of this gene set suggested that peroxisome proliferator activated receptor (PPAR) nuclear receptors regulated aldosterone production and altered signalling in the female KO mouse, which could explain the reduced aldosterone secretion. We tested this hypothesis in H295R adrenal cells and showed that the selective PPARα agonist fenofibrate can stimulate aldosterone production and induce Cyp11b2. Dosing mice *in vivo* produced similar results. Together our data show that *Kcnj5* is important for baseline aldosterone secretion, but its importance is sex-limited at least in the mouse. It also highlights a novel regulatory pathway for aldosterone secretion through PPARα that may have translational potential in human hyperaldosteronism.

## Introduction

Aldosterone plays a central role in blood pressure regulation, and its dysregulation is seen as increasingly important in hypertension. Up to 10% of hypertension is now attributed to primary aldosteronism (PA), characterized by autonomous aldosterone production, independent of the renin–angiotensin pathway [1]. Recent guidance suggests that a third of PA patients have unilateral aldosterone producing adenomas (APAs) in an adrenal gland [1]. Exome sequencing has also shown that over a half of these APAs harbour somatic mutations in the potassium channel encoding gene, inwardly rectifying K channel CNJ type (*KCNJ*) (*KCNJ5*) [2–4]. The importance of these *KCNJ5* mutations in PA has been reviewed in detail [5,6].

The prevalence of *KCNJ5* mutations in APAs varies between cohorts, with up to 70% of APAs in some south Asian cohorts harbouring *KCNJ5* mutations [7]. *KCNJ5* encodes a G-protein regulated inwardly rectifying potassium channel (GIRK4), with the majority of mutations identified in APAs affecting the cation selectivity of this channel, and resulting in increased Na^+^ permeability [2,8,9]. The current consensus posits that this increased Na^+^ permeability of the mutant GIRK4 allows Na^+^ influx into the normally hyperpolarized aldosterone-producing cells of the zona glomerulosa (ZG) causing them to depolarize [4]. This depolarization opens voltage-gated calcium channels that activate Ca^2+^/calmodulin-dependent protein kinases, increasing transcription of aldosterone synthase (*CYP11B2*) and eventually increasing aldosterone production.

Despite extensive characterization of the *KCNJ5* mutations identified in APAs, little is known about the role or importance of the wild-type (WT) GIRK4 channel in aldosterone regulation. Yet, *KCJN5* is expressed at higher levels in the adrenal than the atria (http://www.gtexportal.org/home/gene/KCNJ5) where its role in the muscarinic currents in the heart is well understood. This differential expression of transcript in the adrenal is also seen for other potassium channels such as the two-pore K channel (K2P) TASK1 (KCNK3) channel (http://www.gtexportal.org/home/gene/KCNK3). The ZG cells have been shown to have a resting membrane potential of approximately −80 mV, close to the E_k_ of potassium (−90 mV) in these cells, and TASK channels are thought to be important contributors to the high resting K permeability of rodent ZG cells [10,11]. WT GIRK4 channels, probably as heterotetrameric channels with *KCNJ3*, could contribute to the basal hyperpolarization of the ZG cell. Equally they may have a role in ZG repolarization after they are depolarized in response to ATII, since the Gβγ subunits liberated activate GIRK4 channels [12,13]. This would suggest an inhibitory role for the channel, resulting in the ZG cell requiring a larger depolarizing stimulus for aldosterone production.

By utilizing the previously established *KCNJ5* (GIRK4) *(^−/−^)* knockout (KO) mouse line [14,15], we have investigated the role of WT GIRK4 in the mouse adrenal and its impact on aldosterone secretion.

## Methods

### Animals and tissue collection

#### *Kcnj5* (^−/−^) KO mice

They were a generous gift from Dr Kevin Wickman (Department of Pharmacology, University of Minnesota, Minneapolis, MN, U.S.A.) and Dr Matteo Mangoni (Centre National de Recherche Scientifique (CNRS UMR 5203), Department of Physiology, Montpelier, France) and were maintained in Cambridge by outcrossing with WT C57/BLJ6 mice that were also used as the littermate controls, animals were used for experiments aged 13–16 weeks [14,15]. The *KCNJ5* genotype of each mouse used was confirmed by PCR: neomycin primers 5′ ATGGATTGCACGCAGGTT 3′, 5′ GATACCGTAAAGCACGAGGAAG 3′; coding exon 1 (exon 3 modern mRNA), 5′ TAGAACCACAGGACACCTAGTGAG 3′, 5′ CATTGCCTACGGACGGG 3′. The animal research was regulated under U.K. law, specifically the Animals (Scientific Procedures) Act 1986 Amendment Regulations 2012 following ethical review by the University of Cambridge Animal Welfare and Ethical Review Body.

### Immunohistochemical staining

Formaldehyde fixed paraffin embedded (FFPE) samples were cut using a microtone to 5-μM sections. Sections were deparaffinized in histoclear II (National Diagnostics, Atlanta, GA) and dehydrated in graded ethanol ending in ddH_2_O. Antigen retrieval was performed using standard procedure in the 2100-Retriever (http://www.aptum-bio.com) using commercial universal antigen retrieval solution (http://www.aptum-bio.com).

Mounted tissue sections were stained using the Envision DAB enhancer kit from Dako following manufacturer’s protocol with anti-DAB2 (disabled 2) (http://www.bdbiosciences.com). The following commercial antibodies were used:

**Table:** 

Target	Manufacturer	Catalogue number	Dilution
KCNJ5	Alomone	APC-027	WB 1/200
			IHC 1/20
KCNJ5	Sigma	HPA017353	WB 1/250
			IHC 1/100
KCNJ5	Santa Cruz Biotechnology	A-14	WB 1/200
KCNJ5	Santa Cruz Biotechnology	H-60	WB 1/200
KCNJ5	Abcam	ab125099	WB 1/200
DAB2	BD Biosciences	610464	IHC 1/1000
			LCM 1/500

### Laser capture microdissection

An optimized immunohistochemical protocol using the Envision Plus IHC kit from DAKO was used to stain DAB2. In brief, FFPE 5 μM sections mounted on to slides were rapidly rehydrated through Histoclear II and reducing ethanol solutions, each for 20 s. Antigen retrieval was performed at 60°C overnight with universal antigen retrieval solution (Aptum Biologicals, https://www.proteogenix-products.com), peroxidases blocked for 10 min, and both primary (anti-DAB2) and secondary antibodies incubated for 10 min each. Between each step, slides were washed twice for 1 min in PBS. Following staining, slides were rapidly dehydrated in ascending ethanol solutions for 20 s each, ending with 100% ethanol, allowed to dry and used immediately for laser capture. Laser capture was performed on a Leica LMD6, with samples falling directly into lysis buffer before immediate extraction using the Qiagen RNeasy FFPE kit (https://www.qiagen.com) according to manufacturer’s instructions.

### Plasma collection and electrolyte measurement

Animals were anaesthetized under 3% isoflourane in O_2_ and a terminal blood sample collected by cardiac puncture; blood was immediately transferred to lithium heparin coated tubes for plasma collection. This fresh blood was also analysed on the iSTAT using EC8+ cartridges (www.pointofcare.abbott). Plasma was removed, snap frozen and stored at −80°C.

### Aldosterone assay

Aldosterone was measured from 10 μl frozen plasma using the Cisbio aldosterone assay kit according to manufacturer’s instructions (www.cisbio.com), in which charcoal-stripped serum was used for dilution of the assay standards.

### RNAseq sample preparation and sequencing

Total RNA, including miRNA was extracted using Qiagen miRNeasy kit from 16 whole mouse adrenals taken from animals between 13 and 16 weeks of age, four from each group; male WT (^+/+^), male KCNJ5 (^−/−^), female WT and female KCNJ5 (^−/−^). Quality of extracted RNA was analysed using an Agilent TapeStation (www.genomics.agilent.com), all RNA samples reached RIN numbers >9. The RNAseq analysis was performed by Cambridge Genome Services (www.genomicservices.path.cam.ac.uk) using Ilumina Mouse WG6 beadchips (approximately 45000 markers mapped to NCBI RefSeq build 36.2, release 22) and analysed using the EdgeR Bioconductor package (https://bioconductor.org/packages/release/bioc/html/edgeR.html). Pathway analysis of the differentially expressed genes was performed with the Ingenuity Package Analysis (IPA®) software package (https://www.qiagenbioinformatics.com/products/ingenuity-pathway-analysis/). The RNAseq data used in this manuscript have been publically deposited on GEO.

### H295R culture and treatments

H295R cells were maintained as described previously [16]. Cells were treated with the described drugs or DMSO as vehicle control for 24 h after plating in 96-well plates. The following drugs were used: rosiglitazone (www.sigmaaldrich.com R2408), fenofibrate (www.sigmaaldrich.com F6020) or GW 6471 (www.sigmaaldrich.com G5045). For the last 24 h of treatment, cells were also treated with 10 nM angiotensin II (Ang-II) (www.sigmaaldrich.com, A9525) ± 10 nM losartan (www.sigmaaldrich.com 61188). At the final time point, medium was removed for aldosterone quantification and cell viability measured by MTT assay as described previously [16]. Aldosterone production was normalized by viability within each well. For gene expression experiments, medium was discarded and cells lysed for RNA extraction using Purelink RNA extraction kit (www.thermofisher.com).

### Gene expression

Reverse transcription was carried out using Superscript IV (www.thermofisher.com) as per manufacturer’s protocol. Gene expression analysis was carried out using the TaqMan® Fast Advanced Master Mix and validated TaqMan® Gene Expression Assays according to manufacturer’s protocol (www.thermofisher.com) on an ABI 7500 platform. Probes used: mouse CYP11B2, FAM-MGB, Mm01204955_g1, mouse KCNJ5, FAM-MGB, Mm01175829_m1 and eukaryotic 18S, VIC-MGB, 4319413E. Relative gene expression was calculated by the Δ*C*_T_ method against 18S as the housekeeper and expressed as a multiple of the control value (set to 1) [17].

### Statistics

Normally distributed data were analysed by ANOVA with post-hoc testing or Student’s *t* tests as appropriate using Prism 6 software (www.graphpad.com). Significance was taken as *P*<0.05.

## Results

### Adrenal morphology of *Kcnj5* (^−/−^) KO mice is unchanged

**Figure 1:**
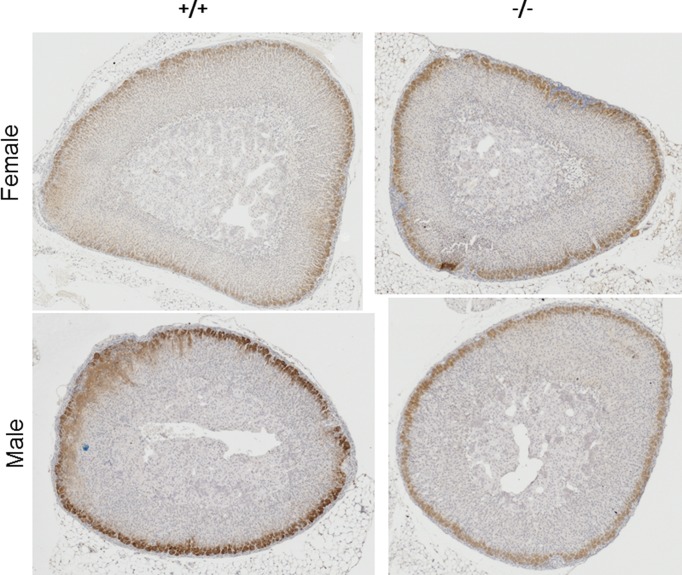
Representative sections of male and female WT (^+/+^) and KO (^−/−^) mouse adrenals stained for DAB2

There were no obvious macroscopic differences between the adrenal glands recovered from KCNJ5 KO (^−/−^) compared with WT (^+/+^) mice. Sections of the glands also showed that zonation between the cortex and medulla (M) was maintained. Using the specific disabled 2 (DAB2) marker [18], the ZG also had a similar depth in both KO (^−/−^) and WT (^+/+^) glands (Figure 1 below).

### *Kcnj5* is specifically expressed in the mouse ZG

We next determined the expression and localization of *Kcnj5* in the adrenal gland of WT (^+/+^) C57BL/6 mice. Due to the lack of a commercially suitable antibody with specificity for the channel in the mouse (Supporting Data), we carried out *Kcnj5* gene expression analysis by qPCR of WT (^+/+^) mouse adrenal tissue. The specificity of the *KCNJ5* gene expression assay was confirmed by gene expression analysis in WT (^+/+^) and KCNJ5 KO (^−/−^) brain and adrenal cDNA (Supporting Data).

**Figure 2:**
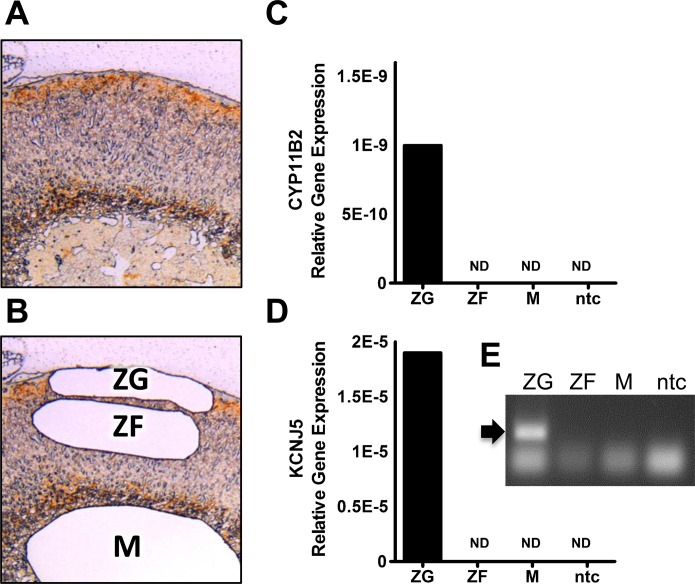
YP11B2 and KCNJ5 gene expression in laser captured adrenal zones (**A**) DAB2-stained adrenal section before (A) and after (**B**) laser capture of specific adrenal zones. Relative gene expression of *Cyp11B2* (**C**) and KCNJ5 (**D**). Bars are means of two experiments in laser captured tissue. (**E**) (Inset) shows gel separation of qPCR products for (D). Abbreviations: ND, not detected by 45 cycles; ntc, no template control.

To confirm that *Kcnj5* was specifically expressed in the outer ZG, as in the human adrenal cortex, laser capture microdissection was used to recover tissue from the ZG, zona fasciculata (ZF) and M. We confirmed successful microdissection by examining *Cyp11B2* gene expression as a specific marker for the ZG (Figure 2 below). This showed that only cDNA extracted from the ZG expressed both *Cyp11B2* and *Kcnj5* genes, confirming that in the mouse *Kcnj5* is expressed specifically by the aldosterone-producing cells of the ZG.

### Female *Kcnj5* (^−/−^) KO mice display lowered plasma aldosterone levels

**Figure 3:**
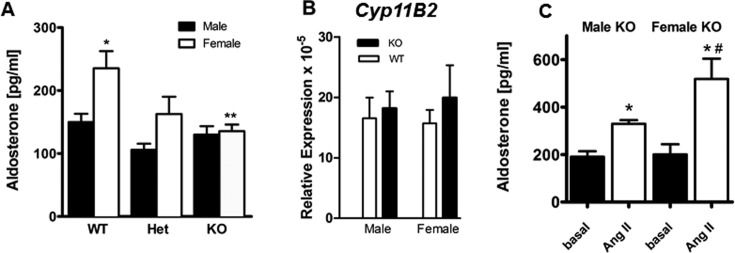
Differences in basal and Ang-II stimulated aldosterone levels and adrenal CYP11B2 gene expression in mice by genotype and sex. (**A**) Basal plasma aldosterone levels in male and female KO (KCNJ5^−/−^), heterozygous (KCNJ5^+/−^) and WT (KCNJ5^+/+^) mice (*n*=7). *P<0.05 compared with male WT, **P<0.05 compared with female WT. (**B**) Levels of *Cyp11B2* expression in WT compared with KO male and female mice (*n*=4). (**C**) Levels of plasma aldosterone before and 30 mins after Ang-II in male and female KO (KCNJ5^−/−^) mice (*n*=4–7). *P<0.05 compared with basal, ^#^*P*<0.05 male compared with female KO. Bars are mean ± S.E.M.

We measured plasma aldosterone levels in male and female adult KCNJ5 KO (^−/−^), heterozygous (^+/−^) and WT mice (^+/+^), both at baseline and 30 min after an Ang-II challenge (200 mg/kg IP). Female WT mice displayed significantly higher plasma aldosterone levels than age-matched WT males (Figure 3). Basal plasma aldosterone levels were also unchanged across genotypes in male mice (Figure 3A below). In contrast, female *Kcnj5* KO animals had significantly lower basal plasma aldosterone levels than female WT littermates. The levels in heterozygous females were intermediate. These differences were not explained by differences in *Cyp11B2* gene expression (Figure 3B below). Aldosterone levels after Ang-II challenge were significantly higher in female compared with male KO mice, despite having similar basal levels of aldosterone (Figure 3C below).

### Electrolytes

**Figure 4:**
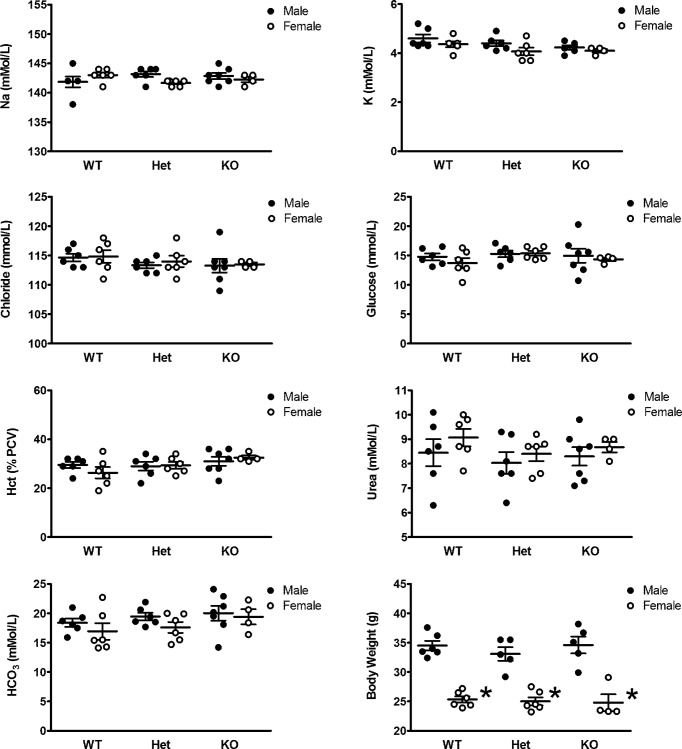
iSTAT blood and haematocrit measurements and body weight for WT (^+/+^), heterozygous (^+/−^), and KO (^−/−^) mice by sex The horizontal lines are mean ± S.E.M. for *n*=4–7 mice. *P<0.001 male compared with female.

Blood electrolytes in male and female *Kcnj5*-deficient mice were measured in fresh whole blood using an iSTAT device. The blood levels of K showed a downward trend from WT to homozygous KO and were consistently lower in females compared with males. However, neither of these trends were statistically significant. No differences were observed between sexes or genotypes in blood levels of sodium, chloride, glucose, urea, HCO_3_ or percentage haematocrit. There was also no significant difference in body weight between genotypes, although males were significantly heavier than females throughout (Figure 4).

### RNAseq reveals major sex differences in the mouse adrenals

To explore the molecular basis for the sex differences in aldosterone secretion, we carried out RNAseq analysis of WT and *Kcnj5* (^−/−^) KO adrenals from both male and female mice. The RNAseq results showed that many more genes were differentially expressed in females compared with males (Table 1 below).

**Table 1:** Number of genes differentially expressed in WT compared with KCNJ5 (^−/−^) KO adrenals from male and female mice (*n*=4)

Analysis (WT compared with KO)	*P*<0.05 (and logFC >±2)	FDR <0.05 (and logFC >±2)
Male	285 (21)	7 (5)
Female	1470 (122)	359 (25)

The pattern was the same whether the significance threshold was pairwise or the more stringent false discovery rate (FDR). The logFC is the log to base 2 of the fold-change and numbers in brackets are for genes that are differentially expressed, at least four fold.

The five differentially expressed genes in the male did not include any genes plausibly linked to aldosterone production (Supplementary Data online). Of note, *Kcnj5* was itself overexpressed in both male and female KO adrenals (Supplementary Data online). However, the Sashimi plot (Supporting Data) of the RNAseq data showed that exon 3 of *Kcnj5* was not expressed in keeping with its deletion and replacement with a neomycin resistance gene (Neo^r^) in the deletion cassette. This explains our failure to detect a full-length *Kcnj5* cDNA on qPCR of the *Kcnj5* KO (^−/−^) adrenals.

**Figure 5:**
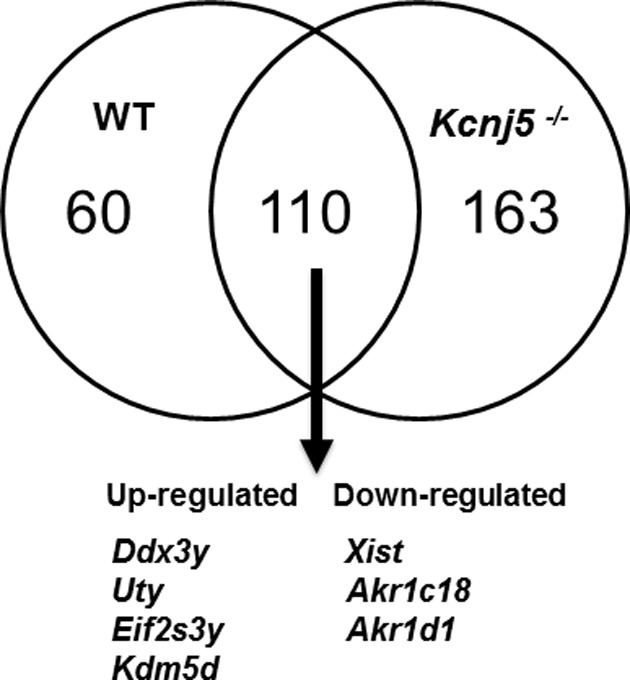
Venn diagram representation of the differentially expressed genes (female compared with male) in adrenals from WT and KO (KCNJ5^−/−^) mice The top seven genes in both gene lists are identical.

We then compared the genes differentially expressed in male compared with female WT mice and those expressed in male compared with female KCNJ5 (^−/−^) KO adrenals. This identified 184 genes that were shared between WT and *Kcnj5* (^−/−^) KO adrenals (Figure 5, below). In both analyses, sex-specific genes encoded on the X and Y chromosomes (*Uty, Eif2s3y, Kdm5d* and *Xist*) were ranked among the top ten differentially regulated genes (Supplementary Data). Two aldo-keto reductases *Akr1c18* and *Akr1d1* were down-regulated in females of both genotypes compared with their male counterparts, and the potassium channel encoding gene *Kcnk1* had a consistently lower gene expression in males compared with females of the same genotype. There were also a number of differentially expressed genes unique to each genotype, including the Y chromosome gene *Ddx3y*, that was only differentially expressed in WT adrenals.

### Pathway analysis of differentially expressed genes suggests that PPAR pathways regulate aldosterone secretion

To look for functional connections between the differentially expressed genes in the female adrenals, we undertook pathway analysis using the Ingenuity® software package (Ingenuity Pathway Analysis (IPA)) using the genes differentially expressed (log_2_FC >±1.5) between female WT and *Kcnj5* KO (^−/−^) adrenals. This identified five canonical pathways and upstream regulators that highlighted a major role for the RXR nuclear receptor in controlling adrenal function (Table 2 below). RXR is in turn regulated by forming heterodimers with peroxisome proliferator activated receptor (PPAR) α and PPARγ. This analysis also predicted that the pathways through these nuclear receptors were uniformly down-regulated in the KO female adrenals.

**Table 2:** Top regulated canonical pathways and upstream regulators from Ingenuity analysis of the genes differentially expressed between female WT and KCNJ5 KO (^−/−^) adrenals

Canonical pathway name	*P*-value	Overlap
FXR/RXR activation	1.58 × 10^−10^	11.7% 16/137
L×R/R×R activation	5.69 × 10^−10^	11.7% 15/128
TR/R×R activation	4.11 × 10^−9^	12.4% 13/105
Retinol biosynthesis	3.11 × 10^−8^	18.4% 9/49
Acute phase response signalling	2.13 × 10^−7^	8.2% 14/171
**Upstream regulator**	***P*-value of overlap**	**Predicted activation**
TO-901317 (L×R agonist)	1.66 × 10^−36^	Down in KO
PPARα	2.89 × 10^−36^	Down in KO
PPARγ	1.15 × 10^−35^	Down in KO
Rosiglitazone (PPARG agonist)	3.16 × 10^−33^	Down in KO
BSCL2	7.87 × 10^−29^	-

Ranked by *P*-value.

### Activation of PPARα but not PPARγ leads to Ang-II-independent aldosterone production in H295R cells

**Figure 6:**
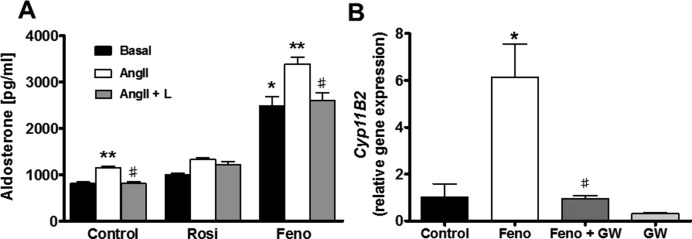
Aldosterone production by cultured H295R adrenal cells exposed to PPAR agonists (**A**) Aldosterone production from H295R cells treated with either control (0.1% DMSO) or 10 μ PPAR agonists: rosiglitazone (Rosi, PPARγ) or fenofibrate (Feno, PPARα) for 48 h. Cells were also treated for the final 24 h with 10 nM Ang-II or Ang-II with 10 nM losartan (Ang-II + L). Error bars represent S.E.M. of *n*=4. *P<0.05 compared with basal control, **P<0.05 compared with basal of same treatment,^ #^*P*<0.05 compared with Ang-II of same treatment. (**B**) *CYP11B2* gene expression in H295R cells treated for 24 h with either control (DMSO), 10 μM fenofibrate, 10 μM fenofibrate with GW6471 or 10 μM GW6471 alone. Error bars represent S.E.M. of *n*=4. *P<0.05 compared with control, ^#^*P*<0.05 compared with Feno.

IPA of the adrenal RNAseq data suggested that PPARα- and PPARγ-mediated pathways were involved in regulating adrenal aldosterone production. To test this *in vitro*, we exposed adrenal H295R cells to PPAR pathway agonists. The PPARγ agonist, rosiglitazone (10 μM), produced no significant change in aldosterone production from H295R cells treated for 48 h with the drug (Figure 6, below). However, rosiglitazone effectively blocked aldosterone production stimulated by 10 nM Ang-II for 24 h, suggesting that PPARγ activation may inhibit Ang-II-mediated aldosterone production. In contrast, cells treated with the PPARα agonist, fenofibrate (10 µM), showed a significant Ang-II independent increase in aldosterone production. This increase in aldosterone production was accompanied by a significant elevation in *Cyp11b2* gene expression after 24 h fenofibrate treatment, which could be reversed by addition of the specific PPARα antagonist GW6471.

### PPARα activation causes aldosterone production in female mice

**Figure 7:**
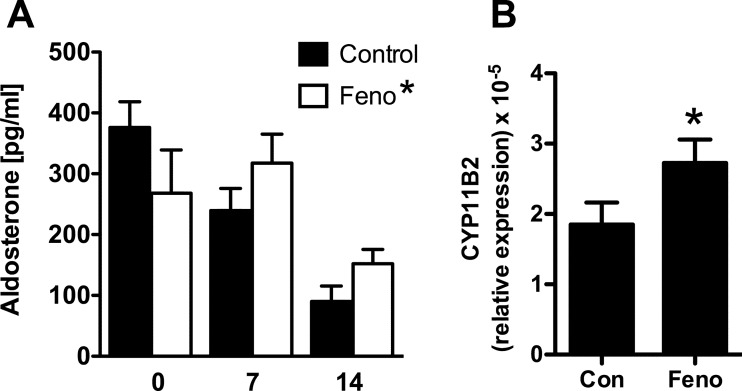
Effect of in vivo administration of fenofibrate on plasma aldosterone levels and adrenal CYP11B2 gene expression (**A**) Effect of 7 or 14 days administration of fenofibrate (100 mg/kg PO) or vehicle (olive oil) on plasma aldosterone levels in female WT mice. *There is a significant treatment effect on two-way ANOVA (*P*=0.003). (**B**) Expression of CYP11B2 in the adrenals after 14 days of dosing with fenofibrate (Feno) or vehicle, *P<0.05 compared with control. Bars are mean ± S.E.M. for *n*=4.

To see if the *in vitro* effect of PPARα activation on aldosterone production could occur *in vivo*, WT female mice were treated with fenofibrate (100 mg/kg PO) or vehicle (olive oil) daily for 2 weeks. Mice that received fenofibrate showed a significant increase in plasma aldosterone levels compared with control treated mice, but the effect was masked by the effect of the olive oil vehicle on aldosterone production (Figure 7, below). There was also a modest increase in *Cyp11B2* gene expression in adrenals from fenofibrate treated mice compared with control (Figure 7). Together this supported the *in vitro* finding in H295R cells, showing that activation of PPARα by fenofibrate leads to increase in aldosterone production.

## Discussion

Interest in the role of the GIRK4 K channel in controlling adrenal aldosterone production has emerged only in the last few years with the discovery that both germline and somatic mutants in the *KCNJ5* gene cause hyperaldosteronism [2,5]. However, there are no data on the role played by the WT GIRK4 channel in the normal adrenal. Our studies reported here show that female but not male *Kcnj5*-deficient mice have lowered plasma aldosterone levels compared with WT counterparts. This finding is mirrored by the observation of transcriptional changes in the female *Kcnj5* (^−/−^) KO adrenal, which are not present in male *Kcnj5* (^−/−^) KO adrenals. IPA analysis indicated that the pattern of differentially expressed genes in the female KCNJ5 KO compared with WT adrenals implicated PPARα and PPARγ pathways in the regulation of aldosterone secretion. We have confirmed the role of PPARα by demonstrating that activation of PPARα with fenofibrate, a specific agonist for this nuclear receptor, increases aldosterone production both in adrenal H295R cells and mice.

In the present study, there were gender differences in plasma aldosterone with the female having significantly higher levels than age-matched males. Even larger female over males differences in plasma aldosterone were reported in the C57/BL6J strain by Heitzmann et al. [19]. In parallel with these differences in aldosterone levels, we have identified major differences in the adrenal transcriptome of the female compared with male mice. Sexual dimorphism within the renin–angiotensin system is well documented and gender differences in WT adrenal gene expression was first reported by El Wakil et al. [20]. They found some 269 genes that were differentially expressed in male compared with female WT mouse adrenals. Other studies have also reported sex differences in adrenal phenotype, morphology and gene expression in both inbred strains and genetically modified mice [21–23]. For example, a microarray analysis of *KCNK3* (TASK1) KO (^−/−^) mice showed differences in gene expression and phenotype between female and male mice, with castrated male mice having a similar gene expression profile to sham-operated female mice [24].

We found that like human ZG mRNA for *KCNJ5* was readily detected in the ZG of the mouse, although it has been reported that *KCNJ5* mRNA was undetectable in the rat adrenal cortex (ZG or ZF) [25]. Chen et al. [25] also failed to show staining for KCNJ5 protein. However, in our hands, the antibody they used did not give specific staining for KCNJ5 protein in either IHC sections or Western blots from the mouse adrenal cortex (Supplementary Figure S1). Hence, although we could detect KCNJ5 message, the lack of a specific antibody for mouse KCNJ5 meant we could not confirm that protein was also present in the mouse ZG.

A striking feature of the RNAseq experiments was the finding that *KCNJ5* gene products were up-regulated in the KO mice of both sexes. However, the mouse *KCNJ5* mRNA is composed of four exons, but only two code for the mature 419aa protein and the majority of this coding region lies in exon 3. In the KO mice, exon 3 has been swapped for a Neo^r^ coding region, so although the qPCR analysis used a commercial Taqman primer-probe set that maps to the exon 3–4 boundary (Mm01175829_m1, Thermo Fisher), it will only amplify mature mRNA with both coding exons present. This explains our failure to detect a qPCR signal from the KO mice. This was an important negative control, since none of the commercially available antibodies could identify an appropriately sized band for mature GIRK4 protein that was absent from the KO adrenal. It is also notable that other inwardly rectifying K channels were not present among the genes differentially expressed in male or female KO adrenals. It might be expected that loss of GIRK4 activity could be compensated by altered expression of other inward-rectifying K channels. However, we saw no evidence of this in the RNAseq data.

We have identified 170 differentially expressed genes between sexes of our WT adrenals (using a threshold of a four-fold change), and 273 in adrenals from *Kcnj5* (^−/−^) KO mice. Indeed, the top seven genes from both lists actually overlapped between genotypes (Figure 5). Interestingly, of these seven genes, all three genes that were up-regulated in females (*Xist, Akr1c18* and *Akr1d1*) were also up-regulated in the study by El Wakil et al. [20]. This finding confirms the robustness of RNAseq in correctly identifying differentially expressed genes, as we and El Wakil et al. [20] used different platforms yet identified very similar gene lists. Of the down-regulated genes however, only one gene, *Uty*, was consistent between ours and the El Wakil et al. [20] study. However, a large number of other down-regulated genes were consistent between our WT or *Kcnj5* (^−/−^) KO sex comparisons: including *Srd5a2, Hmox1, Acan* and *Susd3*, all of which featured in the top 20 differentially expressed genes in both comparisons (see Supplementary Data). *Srd5a2* encodes type 2 5α-reductase (3-oxo-5-α-steroid 4-dehydrogenase 2), an enzyme responsible for the reduction in keto-steroids, including corticosterone, testosterone and aldosterone to their reduced metabolites. In female adrenals, the aldo-keto reductases, *Akr1c18, and Akr1d1* are highly expressed, and *Akr1d1* encodes steroid 5β-reductase which like 5α-reductase converts keto-steroids into their reduced metabolites. Similarly, the *Akr1c* family of aldo-keto reductases are implicated in progesterone metabolism [26]. The sexual dimorphism in mouse adrenal gene expression is intriguing considering the female predominance in human of APAs expressing *KCNJ5* somatic mutations. A meta-analysis of published data on *KCNJ5* somatic mutations showed 67% of them occurred in females [27–29]. Conceivably, *KCNJ5* mutations could have a greater phenotypic impact on female compared with male adrenal cells because of a greater physiological importance of GIRK4 in the female adrenal. Based on the data presented here, this seems to be the case at least for the mouse. A sex difference in the aldosterone phenotype was also reported in the I*Task1* KO mouse with only female adults displaying hyperaldosteronism [24]. The males were unaffected and appeared to be protected by androgen-regulated up-regulated expression of *Task3* channels. However, it is important to note that H295R cells that are routinely used as an *in vitro* model of human adrenocortical cells and were used in our work are karyotypically female (https://www.lgcstandards-atcc.org/products/all/CRL-2128.aspx). Therefore, sublines derived from H295R such as HAC15 are also female. Hence, there is not an easy cell-based approach to explore the hypothesis that somatic KCNJ5 mutations have a larger effect on aldosterone production if expressed in female compared with male adrenal cells.

Previous work with H295R or HAC15 cells reported that incubation with PPARγ agonists reduced aldosterone production, although reported effects on *CYP11B2* mRNA expression are inconsistent [30,31]. However, we could not reproduce these findings using rosiglitazone (Figure 6). At the PPARγ agonist concentrations previously used (10 μM), we found that cell viability was markedly impaired after 72 h of culture (results not shown), which may explain some of the apparent reduction in aldosterone levels reported beyond 48-h incubation. Our findings with the PPARα agonist fenofibrate are clearer with substantial increases in both aldosterone secretion and *Cyp11B2* mRNA levels. Since the effect is blocked by the selective antagonist GW6471, fenofibrate is selectively acting through PPARα. It has been suggested previously that renin may be under PPARα regulation [32], but stimulation of aldosterone secretion by fenofibrate has not been reported before and was only revealed here from our IPA analysis. The IPA analysis also suggests that the PPARα pathway is deactivated in the KO mouse adrenal, which may explain at least in part why female mice have lower aldosterone levels. Further work is needed to explore the precise mechanism connecting PPARα signalling and aldosterone secretion, but it is clear that this effect is not simply explained by altered RXR or Pparα expression. A more detailed steroid profiling of the female KO mouse or a metabolomics approach may give important clues to the molecular pathways involved.

In summary, we have shown that *Kcnj5* is functionally important for aldosterone secretion in the mouse but it is gender specific. The reduced aldosterone secretion in the female KO mouse may be explained by reduced PPARα signalling, which represents a novel control pathway for aldosterone. Hence, blockade of PPARα signalling may have translational potential in regulating human hyperaldosteronism, although this strategy may be sex limited.

## Clinical perspectives

- Mutations in the the potassium channel, KCNJ5, have been recently identified as a cause of hyperaldosteronism in human hypertension. To further explore the role of KCNJ5 in the adrenal gland, we looked at the phenotypic impact in the KO mouse.
- Female but not male mice with homozygous knock of KCNJ5 have reduced circulating levels of aldosterone. We used RNAseq to probe the molecular basis for this difference comparing WT and KO adrenals. The female adrenal has a much larger set of differentially expressed genes compared with the male (396 compared with 7) and pathway analysis of these genes suggested that PPARα was an important regulator of aldosterone synthesis.
- Our work suggests that KCNJ5 has a role in basal as well as pathological aldosterone secretion, although in the mouse, this basal effect may be sex limited. We have also identified a novel regulator of aldosterone secretion that has translational potential to the human adrenal.

## Supporting information

**Supplemental Figure S1:** Antibody detection of GIRK4 protein. Western blots (A, atria and B, adrenals) of WT and KCNJ5 (^-/-^) KO mouse tissue lysates stained for GIRK4 using commercial antibodies. None of these were able to identify a 70kD band that was absent from the KO tissues. The antibodies used were from: Santa Cruz (A-14); 2. Santa Cruz (H-60); 3. Abcam; 4. Sigma; 5. Alomone. C and D. representative immunocytochemical staining using the Sigma and Alomone antibodies

**Supplemental Figure S2:** Quantitative PCR cycling curves for 18S and KCNJ5 in brain versus KO tissues. In the wild-type tissue the cycling threshold (Ct) was ∼24 for KCNJ5. There was no detectable KCNJ5 signal in the KO tissues even after 40 cycles.

**Supplemental Figure S3:** Sashimi plot of RNAseq data for *KCNJ5* gene locus to show expression level and splicing events in WT and KCNJ5 (^-/-^) KO mouse adrenal RNA. There are no RNAseq sequences in the KO adrenal that map to exon 3 of *KCNJ5*.

**Supplemental Figure S4:** Curve showing relative viability of H295 cells cultured for 72 hrs with PPAR agonists (Rosi, rosiglitazone; Feno, fenofibrate). Data is mean ± SEM for n=3.

**Supplemental Figure S5:** Dose response curves for rosiglitazone and fenofibrate. Aldosterone measurements were made after 48 hours exposure of H295 cells to either drug in the presence and absence of Ang II (10nM). Data is mean ± SEM for n=3.

**Supplemental Table 1:** The five genes differentially expressed in male KO vs WT adrenals (n=4).

**Supplemental Table 2:** Top 10 genes differentially expressed in female KO vs WT adrenals (n=4).

## Abbreviations

Ang-II: angiotensin II
APA: aldosterone producing adenoma
CYP11B2: aldosterone synthase
DAB2: disabled 2
GIRK4: G-coupled inwardly rectifying K channel 4
FFPE: formaldehyde fixed paraffin embedded
IP: intraperitoneal
IPA: ingenuity pathway analysis
KCNJ: inwardly rectifying K channel CNJ type
KO: knockout
M: medulla
PA: primary aldosteronism
PO: oral
PPAR: peroxisome proliferator activated receptor
qPCR: quantitative PCR
WT: wild-type
ZF: zona fasciculata
ZG: zona glomerulosa
