# Supplementary material for: Targeted disruption of the Kcnj5 gene in the female mouse lowers aldosterone levels
Source: Clin Sci (Lond). 2018 Jan 16;132(1):145–56. doi: 10.1042/CS20171285 (PMC6365593; doi:10.1042/CS20171285)
Supplement: Supplementary file 1 [file cs-132-cs20171285_supp1.pdf]

Supplementary Material for Manuscript: Targeted disruption of the KCNJ5 gene in the female mouse lowers aldosterone levels. Hardege et al.

Supplemental Figure S1. Antibody detection of GIRK4 protein. Western blots (A, atria and B, adrenals) of WT and KCNJ5 ($^{-/-}$) KO mouse tissue lysates stained for GIRK4 using commercial antibodies. None of these were able to identify a 70kD band that was absent from the KO tissues. The antibodies used were from: Santa Cruz (A-14); 2. Santa Cruz (H-60); 3. Abcam; 4. Sigma; 5. Alomone. C and D. representative immunocytochemical staining using the Sigma and Alomone antibodies
.

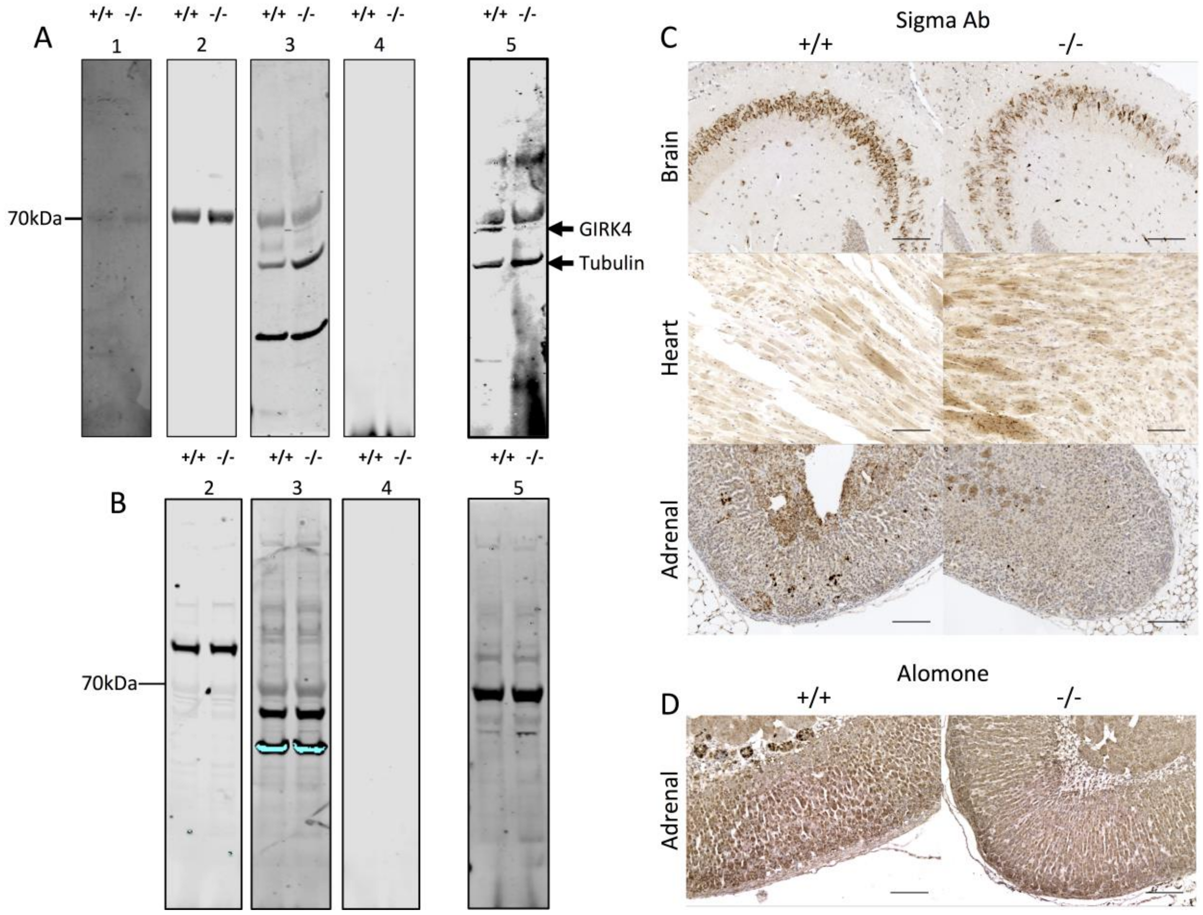

Supplemental Figure S2. Quantitative PCR cycling curves for 18S and KCNJ5 in brain versus KO tissues. In the wild-type tissue the cycling threshold (Ct) was ~24 for KCNJ5. There was no detectable KCNJ5 signal in the KO tissues even after 40 cycles.

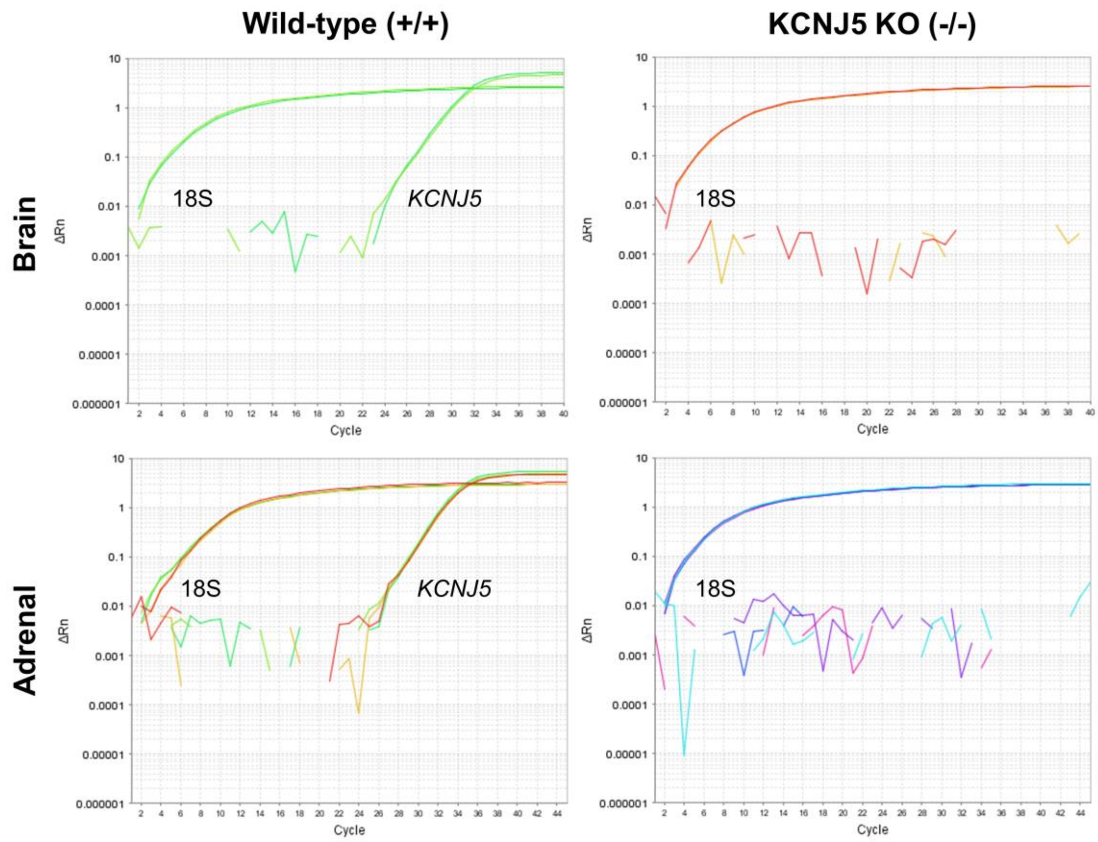

Supplemental Figure S3. Sashimi plot of RNAseq data for *KCNJ5* gene locus to show expression level and splicing events in WT and KCNJ5 ($^{-/-}$) KO mouse adrenal RNA. There are no RNAseq sequences in the KO adrenal that map to exon 3 of *KCNJ5*.

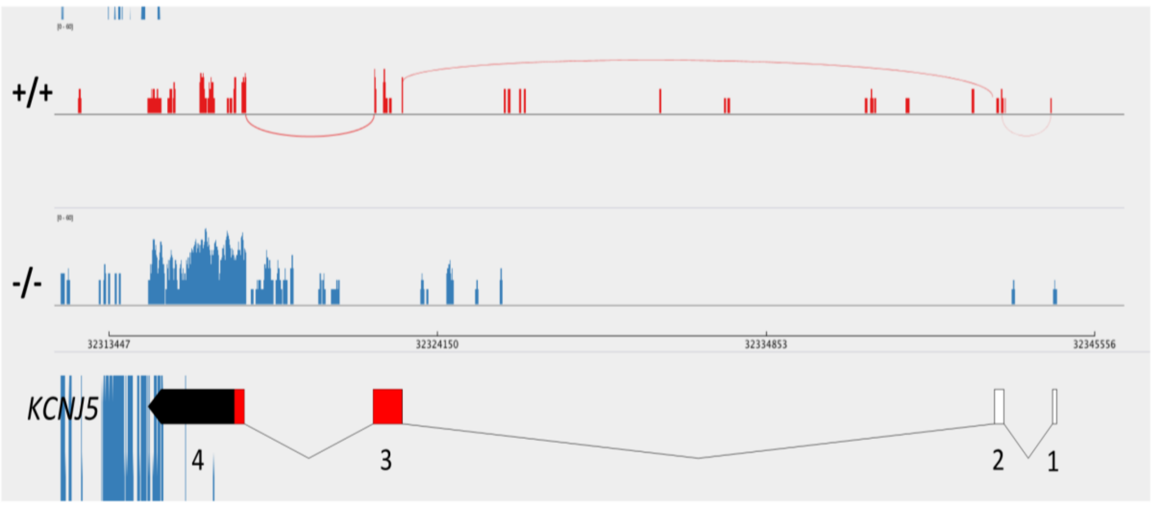

Supplemental figure S4. Curve showing relative viability of H295 cells cultured for 72 hrs with PPAR agonists (Rosi, rosiglitazone; Feno, fenofibrate). Data is mean ± SEM for n=3.

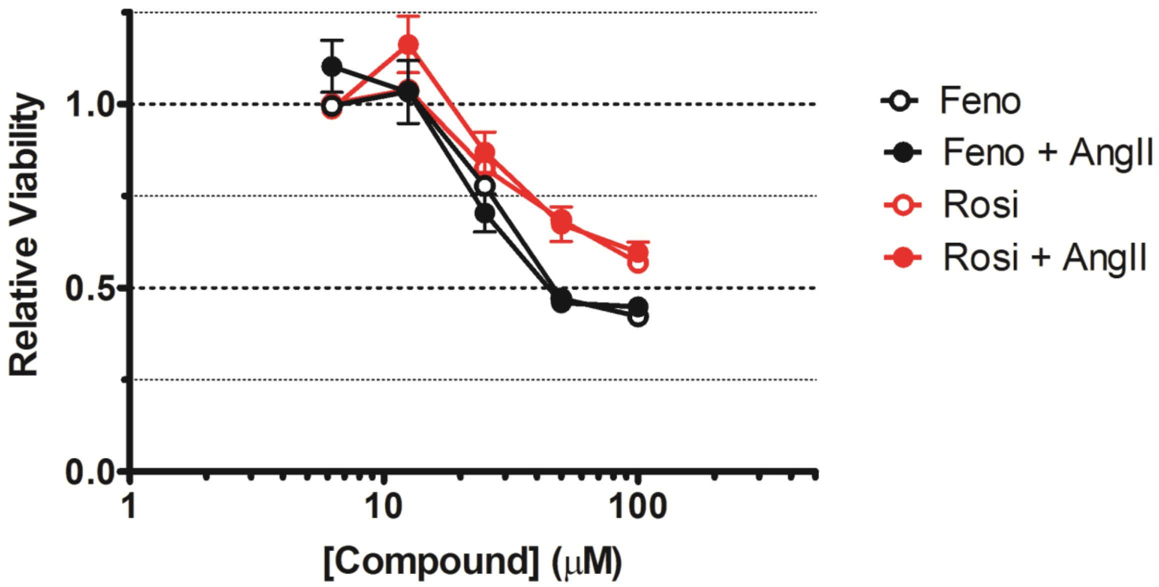

Supplemental figure S5. Dose response curves for rosiglitazone and fenofibrate. Aldosterone measurements were made after 48 hours exposure of H295 cells to either drug in the presence and absence of Ang II (10nM). Data is mean ± SEM for n=3.

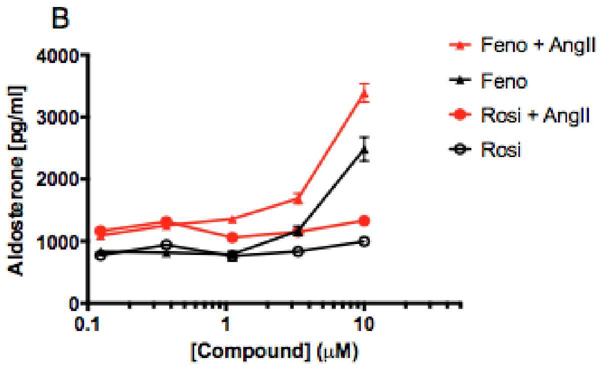

Supplemental figure S5.

Schema to explain the observed aldosterone production in the KO mouse. A. (A). AngII activation of its GPCR receptor depolarizes ZG cells by closing TASK K channels. The $G_{\beta\gamma}$ subunits generated by the AngII GPCR will also open KCNJ5 (GIRK4) channels. This can repolarize the ZG cell switching off aldosterone synthesis triggered by depolarization (B). This suggests KO of the channel may increase not decrease aldosterone production (B and C). However, KO of KCNJ5 in the ZG does not occur in isolation as the RNAseq data shows. The IPA predicts reduced PPARA/G signalling in the KO adrenal, which reduces aldosterone synthesis directly antagonising the impact of loss of the KCNJ5 channel itself (D).

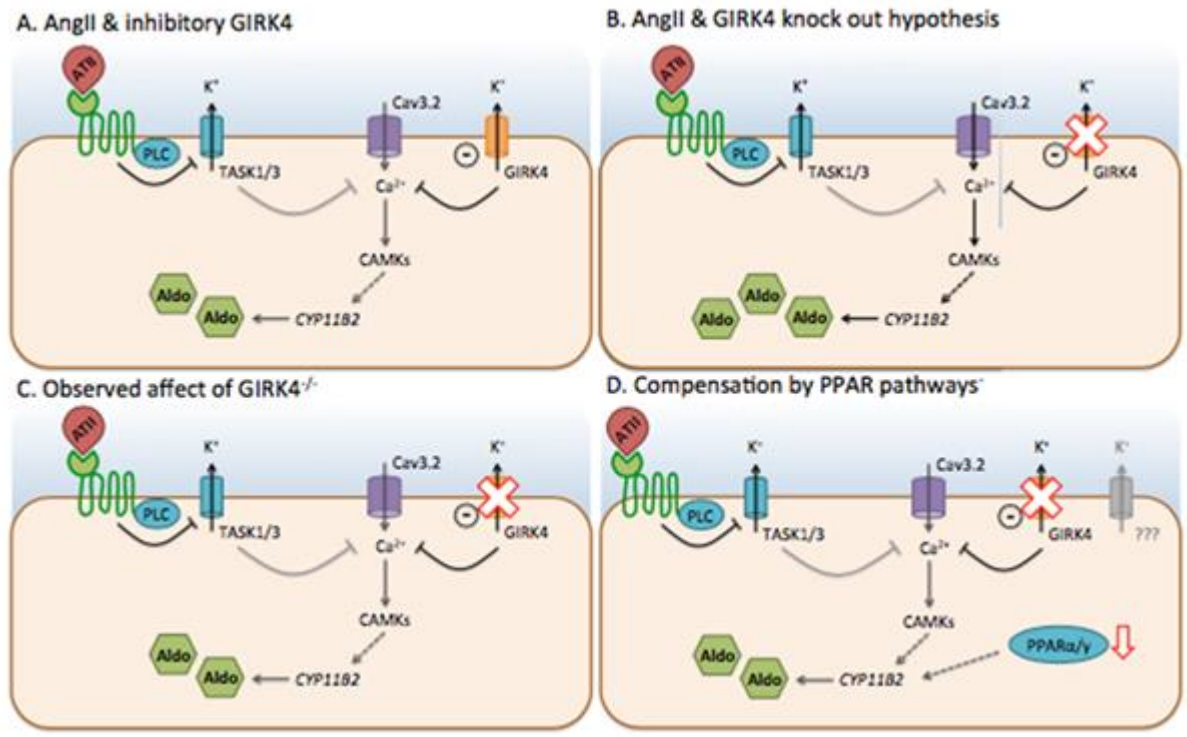

Supplemental Table 1. The five genes differentially expressed in male KO vs WT adrenals (n=4).

Upregulated genes are shown in red and downregulated in blue.

Gene Name	LogFC	P value	FDR	Encoded Protein
KCNJ5	2.22	3.54E-08	3.17E-03	G-Protein regulated inwardly rectifying potassium channel 4 (GIRK4)
Gm10800 (PIRO)	-4.31	2.40E-07	1.43E-02	Five-TM domain-containing receptor-like protein
Tex11	5.09	5.83E-06	0.0261	Testis Expressed
Gm10801	-4.82	7.31E-06	0.0261	Un-annotated
Gm24119	-4.32	1.22E-05	0.036	Un-annotated

Supplemental Table 2. Top 10 genes differentially expressed in female KO vs WT adrenals (n=4).

Upregulated genes are shown in red and downregulated in blue.

Gene Name	logFC	P Value	FDR	Encoded Protein
KCNJ5	2.43	9.35E-25	1.74E-20	G-Protein regulated inwardly rectifying potassium channel 4 (GIRK4)
Serpina5	4.19	1.90E-15	1.18E-11	Alpha-1 Antiproteinase, Antitrypsin
Obp2a	2.56	1.11E-12	1.89E-09	Lipocalin
Gys2	-2.06	9.71E-12	1.21E-08	Glycogen Synthase 2
Col6a4	2.02	8.07E-09	3.49E-06	Collagen, type VI, alpha 4
Esr2	2.87	1.70E-08	6.22E-06	Estrogen Receptor 2
Tdrd5	2.44	1.93E-07	4.92E-05	Tudor Domain Containing 5
Ptprq	3.73	3.35E-07	7.62E-05	Protein Tyrosine Phosphatase, Receptor Type, Q
Foxl2os	3.04	6.00E-07	1.22E-03	Forkhead box L2
Urah	3.01	8.82E-07	1.68E-03	Urate (5-hydroxyiso-) hydrolase

File: Differentially Expressed Adrenal Mouse Genes.xlsx

This is an Excel spreadsheet containing the list of genes that are differentially expressed in the adrenals of WT vs KO mice (defined as a 4-fold change i.e. log2FC ± 2) and have a false discovery rate (FDR) of <0.05. Overexpressed genes are highlighted in red and under-expressed in blue. The comparisons are by genotype (KO vs WT) and sex (M vs F).
